# Creative strengthening groups as a potential intervention to enhance job satisfaction and reduce levels of burnout in healthcare professionals: results from the randomized controlled trial UPGRADE

**DOI:** 10.1186/s12913-025-12644-6

**Published:** 2025-04-17

**Authors:** Claudia Pieper, Melanie Lausen, Desiree Kröckert, Yvonne Klemp, Udo Baer

**Affiliations:** 1 Institute for Medical Informatics, Biometry and Epidemiology, University Hosital of Essen, Hufelandstraße 55, Essen, 45147 Germany; 2 Institute for Social Innovations e.V. (ISI), Blumenstraße 54a, Duisburg, 47057 Germany

**Keywords:** Occupational health promotion, Job satisfaction, Nursing, Healthcare professionals, Prevention, Stress, Burnout, Strengthening, Resilience

## Abstract

**Background:**

Healthcare professionals often face substantial work-related burdens. A large body of evidence has shown that poor working conditions can lead to low levels of job satisfaction, increased emotional stress and burnout. While symptom targeted interventions take effect after symptoms become manifest, preventive interventions are required to reduce the risk of work-related diseases. Therefore, the UPGRADE-trial aimed to evaluate the effectiveness of Creative Strengthening Groups as a potential intervention to enhance job satisfaction and work-related health. The German Innovation Fund (Innovationsfonds) funded the project.

**Methods:**

We conducted a randomized controlled trial and randomly assigned healthcare professionals to either the intervention or the control group. The intervention - Creative Strengthening Groups - consisted of two one-day classes. We evaluated the primary outcome job satisfaction and further work-related outcomes using standardized questionnaires. We collected data at baseline as well as after three and six months. The study was conducted between October 2019 to March 2023, including the pandemic period.

**Results:**

We enrolled 196 participants (intervention *n* = 88, control *n* = 108) with a mean age of 46.2 ± 12.1 years (84.5% female). 43.7% were nursing professionals. Job satisfaction in the intervention group increased from 55.47 ± 10.23 to 57.07 ± 11.65 after three months and decreased in the control group from 56.29 ± 19.69 to 53.47 ± 20.09. The difference between groups did not reach statistical significance. Additionally, change in patient-related stress significantly differed between groups (intervention: -3.9 ± 12.16 vs. control: 5.17 ± 17.43; *p* =.027) as well as personal burnout (intervention: -5.25 ± 13.1 vs. control: 4.35 ± 16.24; *p* =.011). Within the intervention group, we observed a greater improvement concerning work-related burnout in nursing staff in geriatric care than in hospitals (-15.27 ± 13.5 vs. +3.28 ± 13.7; *p* =.003).

**Conclusion:**

Though the Covid-19 pandemic worsened working conditions for healthcare professionals, our results indicate that intervention has the potential to enhance job satisfaction and work related health. Notably, due to the overall workload and the pandemic restrictions, a high number of participants did not continually participate in both classes of the intervention and some did not return all questionnaires. As long as healthcare professionals in Germany are facing exceedingly bad working conditions, it is very difficult to support their resources such as self-efficacy, self-esteem, and optimism by health promotion interventions.

**Trial registration:**

The trial has been registered at the German Clinical Trials Register (DRKS; ID: DRKS00020908). Date of Registration: 2020-03-16.

## Background

Healthcare professionals, especially nursing professionals, face various work-related burdens [1]. These burdens include long working hours as well as night and shift work and physical stress. Together with limited resources, staffing shortages and low salaries, these burdens contribute to poor job satisfaction and low levels of well-being [1, 2]. Again, poor job satisfaction is associated with absenteeism, physical and mental health problems such as burnout, anxiety and depression and psychological stress [1, 3]. In Germany, with an average of 28.8 days of absence in 2022, the nursing staff were around 57% (10.5 days) above the average for all professionals insured by the Techniker Krankenkasse, the largest health insurance fund in Germany [4]. More than one in three nurses (35.2%) have thought about quitting the job in the past 12 months [5].

Regarding the relation of work-related risk factors and well-being among healthcare professionals, Baylina et al. [6] found that psychosocial risk factors have a larger impact on well-being than physical risk factors. Accordingly, Çam and Büyükbayram [7] emphasize the importance of being able to be resourceful and respond to stressful situations that arise in patient care. In intensive care professionals, those with lower levels of resilience showed higher levels of exhaustion, more symptoms of Post-traumatic stress disorder (PTSD) and anxiety and depression [8].

Furthermore, healthcare professionals might experience trauma from workplace violence and the pain and suffering of patients [2, 9–12]. They are at risk of developing PTSD [13–16]. Strikingly, physicians are still underrepresented in PTSD-research, going along with the tabooing of work-related mental health problems of physicians [17]. Workplace violence includes physical violence and non-physical forms of violence such as verbal threats by patients or clients [18–20]. In ambulatory care nursing 61% of professionals report an experience of verbal abuse, 36% experienced physical aggression (such as rough handling, scratching, throwing, or pushing), and one in six reports being sexually harassed within the past year [21, 22]. The prevalence of physically aggressive behavior in dementia patients varies between 31% and 42% [23]. A more recent study indicates an increased number of assaults on healthcare professionals by patients in psychiatric facilities [24]. Hospital nursing staff faces the highest levels of physical violence, followed by nursing staff geriatric care [18]. Yet, 60% of professionals in Germany report not getting enough support from their employers or other professionals when experiencing stressful events [14].

Compared to other occupational groups, factors that cause work-related stress are overrepresented in the healthcare sector [25] and healthcare professionals are more likely to face workplace violence and trauma [18, 26]. Subsequently, this leads to the decision to resign [27], incapacity to work [28] and high staff turnover [29]. Not least, the COVID-19 pandemic put additional stress on healthcare professionals and seriously affected their emotional and psychological well-being [9, 30, 31]. The prevalence rates of anxiety, depression, and insomnia in nurses during COVID-19 were up to 39% [32].

Despite the large evidence, reporting that resilience and inner strength protects against negative psychological outcomes [33, 34], there is a lack of setting-specific interventions and intervention studies in this field taking into account setting-specific health burdens and implementation challenges [35]. Available studies suggest that health promotion interventions for healthcare professionals are likely to have positive effects on job-related stress [36, 37]. Therefore, the development of educational programs and trainings focusing on coping skills and the improvement of resilience and well-being needs to be promoted [15, 33, 38].

There is a growing body of literature supporting creative art therapies in the treatment of mental health problems. Creative art therapy includes painting, dancing and movement, and music therapy, and allows strengthening healthy adult functioning by enabling clients to get in contact with their feelings and needs [39, 40]. Therefore, creative art therapy can stimulate underlying experiences and to encourage clients to find different solutions to a problem.

Based on this knowledge, we designed the UPGRADE trial including an intervention called Creative Strengthening Groups (CSG) for healthcare professionals experiencing high levels of work-related stress. The CSG were designed to strengthen healthcare professionals to identify stressors and to manage stressful situations as well as to rely on their own skills.

The aim of the UPGRADE trial was to evaluate the effectiveness of CSG on job satisfaction and work-related stress. Here we present the participant flow and the results of the randomized controlled trial.

## Methods

### Study population and setting

The target population was healthcare professionals. In 2021, around six million professionals, more than 10% of all employed people, worked in the German healthcare system [41]. The study was conducted in the Rhine-Ruhr metropolitan region, which is the most densely populated region in Germany with approximately 14 million inhabitants.

The intervention was offered to professionals individually or via participating institutions. Participating institutions were healthcare facilities (five hospitals and six of inpatient geriatric care), who agreed to inform the staff about the trial and to release their professionals from work to participate in the intervention.

### Inclusion and exclusion criteria

The inclusion criteria for participants were as follows:

- Working in the healthcare sector (such as nursing staff, medical staff/physicians, therapeutic staff, administrative staff)
- Age of 18 years and older
- Reporting a perceived need for support due to work-related or personal stress overload.

We excluded professionals who concurrently participated in a similar program and those who were under medical treatment because of diagnosed mental health disorder.

### Recruitment

In total, we contacted 94 healthcare facilities via telephone, e-mail, or in person. Additionally, we published information about the trial and the intervention in social media, in press and disseminated flyers to arouse interest among healthcare professionals providing brief information and contact details.

We conducted informative meetings in participating facilities to inform healthcare professionals about the intervention, the study background and to respond questions. Those who were interested in participating received written information and informed consent. Two qualified and appropriately trained members of the research team were responsible for obtaining consent. In obtaining and documenting informed consent, the research team complies with good clinical practice (GCP) and with the ethical principles of the Declaration of Helsinki.

During the Covid-19 pandemic, we conducted informative meetings via video calls and offered a consultation hour for those who were interested to participate. Consent was obtained via fax, e-mail or mail.

Throughout the recruitment phase, we informed professionals who contacted the research team independent of a participating facility personally on appointment, via video calls or telephone.

### Intervention

The UPGRADE intervention was designed by a cooperation of researchers and clinicians from the fields of epidemiology, health promotion and prevention, behavior therapy and psychology.

Based on creative art therapy, the Creative Strengthening Groups (CSG) were designed as two one-day classes for groups of six to ten participants with an interval of two to four weeks [42]. The initial approach provided eight weekly classes of one and a half hour. After consulting employers and professionals of the participating facilitates in September 2021, we adjusted the approach from weekly classes to two one-day classes to make advances to the specific working schedules. The CSG were conducted locally at the participating facilities or at the organizing institute.

The CSG were instructed by a professional therapist, focusing on supporting personal treatment goals, the improvement of cognitive functions, the promotion of self-esteem and self-awareness, and the resolving of conflicts and distress. The aim was to understand factors that cause stress and discomfort and to change them step by step.

The CSG classes involved the use of movement (dance), musical improvisation, painting or drawing as well as mindfulness and self-awareness practices to reflect on emotions without judgment, to acknowledge and accept them and were structured as follows: Introductory phase with welcome, introduction to creative techniques and awareness raising on the respective topic with room for questions. Then the therapist encouraged the participants to exchange experiences about personal and work-related stressors as well as personal strengths. While engaging in a creative activity the facilitator instructed the participants to pay attention with all senses and notice new insights. Once a participant has created a piece of art, for example, they discussed it with the therapist. Talking with a therapist about their creation can help the participants to express their emotions, explore and cope with feelings of distress, sorrow, anger and helplessness related to their experiences. Finally, the participants explained their experiences with the benefits of the CSG and the therapeutic relationship. The schedule was individually adapted to meet the needs of the participants.

During the study period, participants in the control group received “standard treatment”, which means that there was in fact no treatment for the control group, as in normal working life. In case we found a suspected underlying mental disorder or urgent need of support, IG participants were referred to available services such as an occupational therapist.

The intervention was implemented within the framework of a special care contract in accordance with Section 140a SGB V (The fifth book of the Social Code Germany (Sozialgesetzbuch (SGB) Fünftes Buch (V)). Payment was not required.

### Study design

**Fig. 1:**
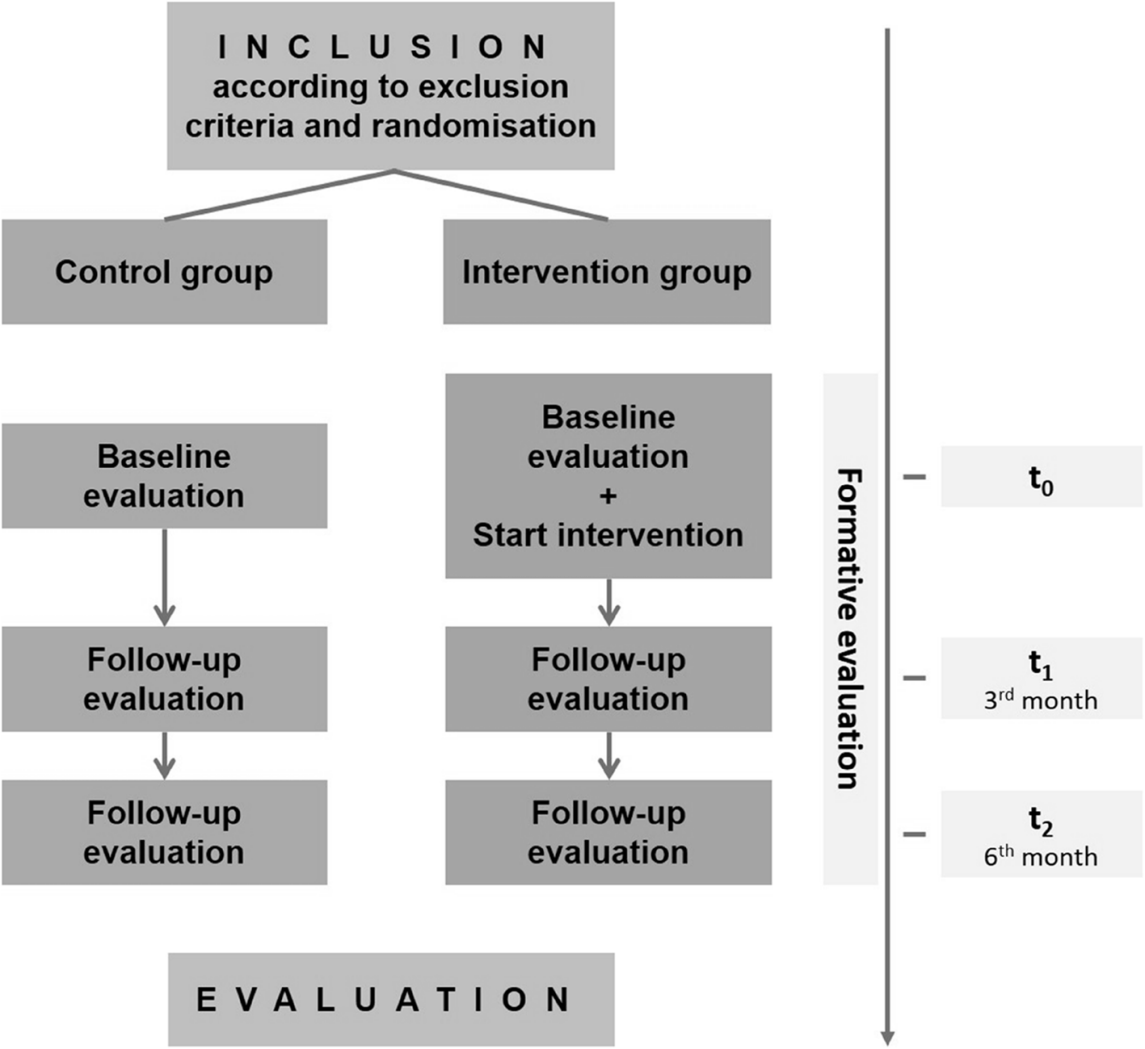
Study design and evaluation concept according to Pieper et al. [42]

The UPGRADE trial is a multi-center randomized controlled trial with two parallel study arms (intervention group (IG) & control group (CG), see Fig. 1). Participants were randomized according an SOP scheme in a 1:1 ratio into either the intervention or the control group between February 2020 and September 2022. As previously described [42] the assignment of participants to treatment groups was kept concealed from both the participant and the researcher until the moment of allocation. Assignment was done by a neutral staff member to avoid any investigator bias. Due to the nature of the intervention, blinding with respect to the intervention itself was not possible.

The study was approved by the lead Ethics Committee of the Medical Faculty of the University of Duisburg Essen (reference number: 19–8995-BO, date of approval: 13/02/2020). All procedures were carried out in accordance with the Helsinki Declaration. The trial has been registered at the German Clinical Trials Register (ID: DRKS00020908).

In order to complement our quantitative analysis, we collected qualitative data by interviewing and observation to gain an understanding of underlying attitudes, opinions and motivations. These results will be addressed in further publication.

### Research question and hypotheses

The main research questions were

1. Are Creative Strengthening Groups effective in enhancing job satisfaction and reducing work-related stress in healthcare professionals?
2. Does the participation in Creative Strengthening Groups improve the perception of specific work-related stresses and strains, ability to work and the general state of health?

Accordingly, we hypothesized that

1. Participants who participated in CSG (IG) report an increase in job satisfaction by 10% relating to the German Copenhagen Psychosocial Questionnaire (COPSOQ) collective (mean value healthcare: 62).
2. Participants who participated in CSG (IG) report an improvement in the perception of specific work-related stresses and strains, ability to work and the general state of health.

### Primary and secondary outcomes and assessments

Data was collected via standardized survey questionnaires, which were offered online- or paper-based. Respondents returned questionnaires by mail using an included pre-paid addressed envelope, or completed them online by following a personal secure survey link.

As job satisfaction being one of the most applied indicator for working conditions and work-related stressors, the primary outcome was difference in job satisfaction after three months (t_1_) as well as after six months (t_2_) relative to baseline (t_0_) between the IG and the CG. Due to the pandemic lockdowns and the extended workload in healthcare during the Covid-19 pandemic, we had to stretch the recruitment period as long as possible. For this reason, t_2_ was not available for those who were included after December 2022. The study design is shown in Fig. 1.

Secondary outcomes were change in perception of specific work-related stresses and strains, work-related, patient-related and personal burnout, work ability and subjective health at baseline and after three (t_1_) and six months (t_2_). Sociodemographic characteristics were collected as moderating variables. Table 1 shows the schedule of interventions and assessments in detail.

We used the German version of the Copenhagen Psychosocial Questionnaire (COPSOQ) [43] version 2018 to evaluate job satisfaction and the perception of specific work-related stresses and strains. The COPSOQ is a validated questionnaire with a current total of 84 items and 31 scales. The job satisfaction scale consists of seven items, including one item overall job satisfaction. We used the Work Ability Index (WAI) [44] to evaluate ability to work, with higher values indicating higher subjective work ability. The Copenhagen Burnout Inventory (CBI) according to Kristensen [45] uses three categories for work- and patient-related stress (“work-related burnout”, “client-related burnout” and “personal burnout”) using scales between 0 (very dissatisfied) and 100 (very satisfied) with high CBI scores indicating high burden. “Physical health status” was measured as mean value of thirteen items [46].

**Table 1:**
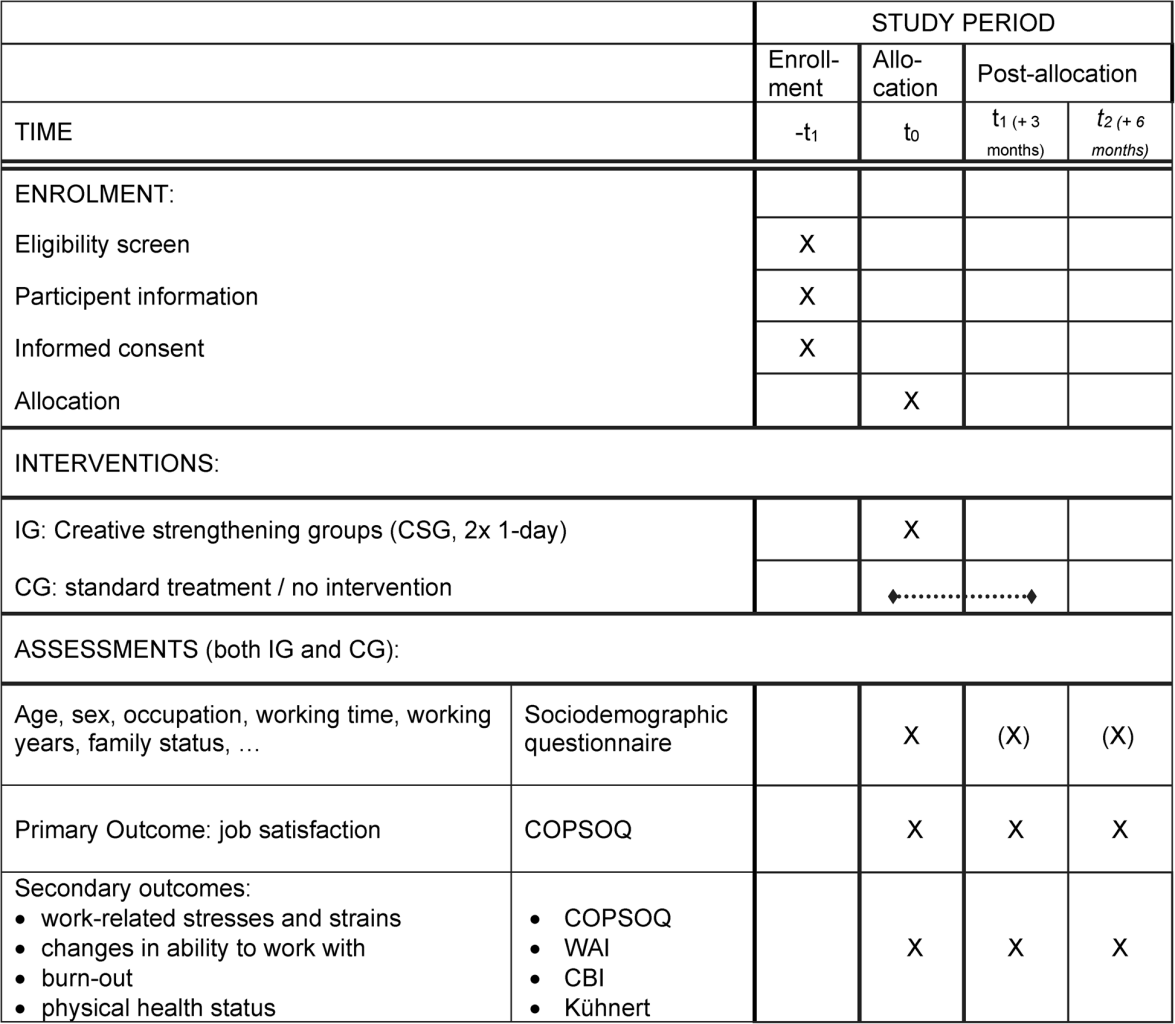
Schedule of enrolment, interventions, and assessments

### Sample size

The sample size calculation was based on the primary outcome “job satisfaction”. Consistent with the German COPSOQ reference values for job satisfaction for healthcare professionals ranging from 61 to 63, we assume an average of 62 (SD = 25). In relation to the hypothesis that participating in the intervention increases job satisfaction by 10% relating to the assumed average of 62, we received a total sample size of *n* = 183 to achieve 80% power (α = 0.05). We increased the initial sample size due to the expected response rate and loss to follow up by 100% to *n* = 366. It is to be considered, that sample size was doubled before the COVID-19 pandemic and at this point, we did not foresee that the pandemic would affect recruitment.

### Statistical analyses

After data entry, we manually and statistically checked collected data as part of the data-cleaning process. Initially, we performed a description of the sample to assess differences between the IG and CG regarding primary outcome and the sociodemographic variables of sex, work status and family status. Following our study protocol, we calculated all standardized scales following the recommendations of the respective scale [23].

We intended to perform the primary confirmatory analysis based on the intention-to-treat (ITT) principle, including all participants randomized regardless of protocol compliance. To assess the effect of the intervention for those who adhered to the protocol (participating in one or two CSG-classes), per-protocol analysis was meant to be performed additionally. Because of the number of missing baseline data, we decided to analyse available data only for those who completed the study endpoints. Missing data was not imputed, because we could not assume that data is missing at random.

Paired t-tests for repeated measures using Cohen’s d were performed to compare the difference in the primary outcome comparing IG and CG. To estimate the associations between the primary outcome and age, sex, work status and further variables we performed a correlation analysis. We used linear regression analysis to describe the relationship between the primary and secondary outcomes and independent variables using R² and Cohen’s f as a measure of effect size. We used SPSS Statistics 29 (IBM Cooperation, 2020) for all analyses. The significance level was set at *p* <.05.

## Results

### Participant recruitment

**Fig. 2:**
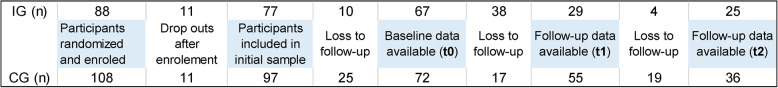
Participant flow

Finally, we enrolled 196 participants (IG: *n* = 88, CG: *n* = 108). In both groups, 11 participants withdrew informed consent. Figure 2 shows the number of participants included in data analysis.

Table 2 shows participation in the CSG units for the IG (regularly two units) and the response to questionnaires for baseline and follow-up for IG and CG.

Participation rate was 51.9% for participating in one or in two CSG units, 43% completed two units (IG).

Response rate was higher in the IG at t_0_ (87% vs. 74.2%) but lower at t_1_ and t_2_. The majority of participants in the IG who did not complete baseline assessment (non-responders) did not participate in the CSG-units. Notably, in the CG 8 participants who completed t_1_ and 4 participants who completed t_2_ did not respond at t_0_ (*n* = 1) and t_1_ (*n* = 3) respectively.

**Table 2:**
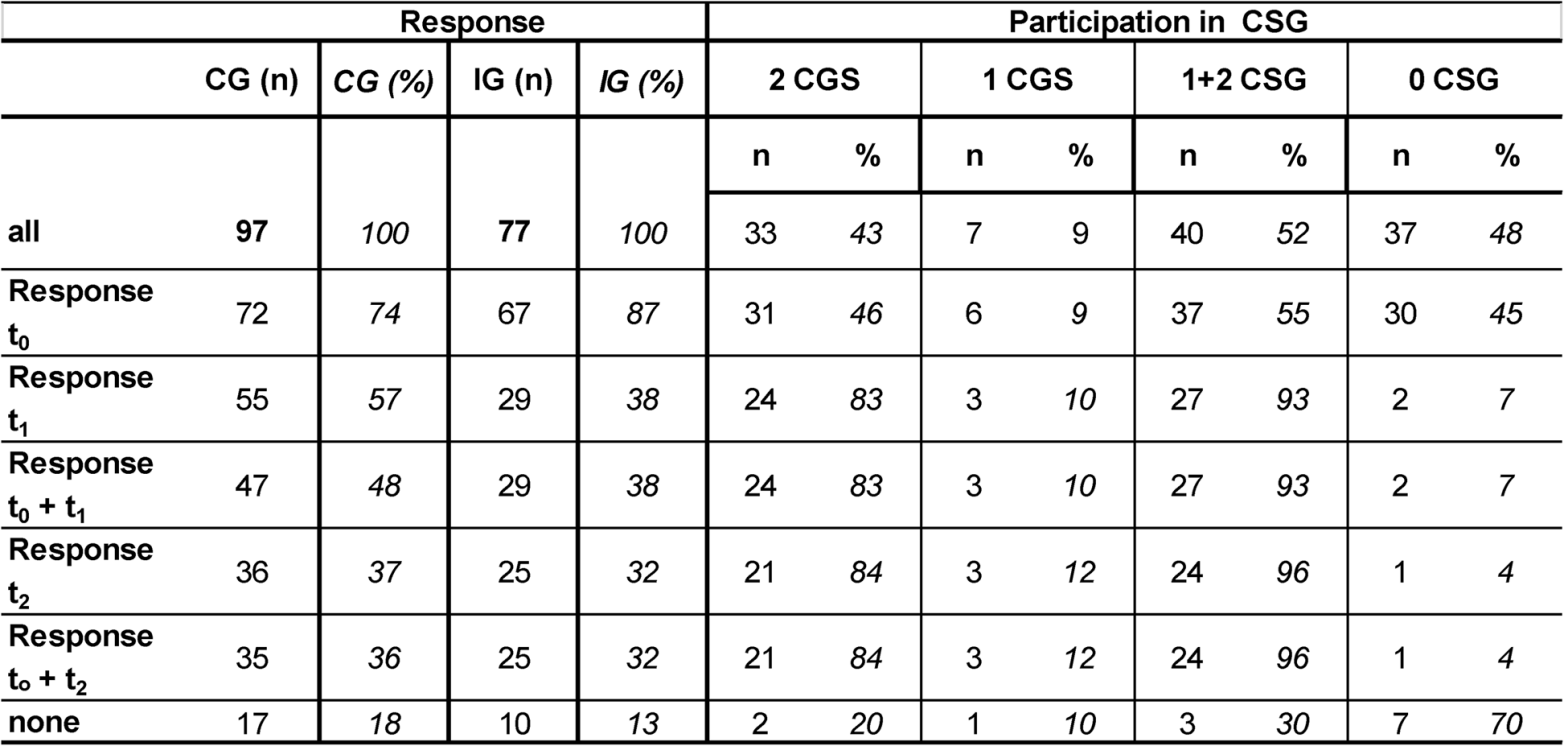
Participation in CSG (IG) and response (return of questionnaires)

### Study population at baseline (t_0_)

In total, 174 participants were included in the analysis, with 139 completing t_0_. Reasons for not completing t_0_ (*n* = 28) were personal reasons (25%) or time constraints and high workload (28.6%).

### Sociodemographic data

Participants were 46.2 ± 12.1 years old, ranging from 18 to 65 years. More than half of the participants were aged between 25 and 49 years, 5.2% were 25 and younger 38.5% were 50 years or older (Table 3). The majority of participants were female (84.5%). In total, 51.7% reported being married or cohabiting. Sociodemographic characteristics did not differ between the IG and the CG.

**Table 3:** Description of the study sample at baseline

		IG (*n* = 77)	IG (%)	CG (*n* = 97)	CG (%)	all (*n* = 174)	all (%)
**Gender**	male	10	*13.0*	13	*13.4*	23	*13.2*
	female	66	*85.7*	81	*83.5*	147	*84.5*
	divers	0	*0.0*	0	*0.0*	0	*0.0*
	n/a	1	*1.3*	3	*3.1*	4	*2.3*
**Mean age (x̄)**	all	45 (± 12.89)		47 (± 11.3)		46.2 (± 12.1)	
**Age in years**	< 25	7	*9.1*	2	*2.1*	9	*5.2*
	25 to 34	8	*10.4*	9	*9.3*	17	*9.8*
	35 to 49	20	*26.0*	24	*24.7*	44	*25.3*
	> 49	32	*41.6*	35	*36.1*	67	*38.5*
	n/a	10	*13.0*	27	*27.8*	37	*21.3*
**Family status**	married	37	*48.1*	37	*38.1*	74	*42.5*
	divorced	5	*6.5*	14	*14.4*	19	*10.9*
	widowed	1	*1.3*	2	*2.1*	3	*1.7*
	cohabited	9	*11.7*	7	*7.2*	16	*9.2*
	single	15	*19.5*	10	*10.3*	25	*14.4*
	n/a	10	*13.0*	27	*27.8*	37	*21.3*
**Caring relatives/ Children**	yes	15	*19.5*	16	*16.5*	31	*17.8*
	no	51	*66.2*	53	*54.6*	104	*59.8*
	n/a	11	*14.3*	28	*28.9*	39	*22.4*
**Working time**	full-time	34	*44.2*	34	*35.1*	68	*39.1*
	part-time	31	*40.3*	37	*38.1*	68	*39.1*
	n/a	12	*15.6*	26	*26.8*	38	*21.8*
**Weekend-working min. once monthly**	yes	41	*53.2*	43	*44.3*	84	*48.3*
	no	23	*29.9*	29	*29.9*	52	*29.9*
	n/a	13	*16.9*	25	*25.8*	38	*21.8*
**Nightshift min. once monthly**	yes	32	*41.6*	33	*34.0*	65	*37.4*
	no	29	*37.7*	34	*35.1*	63	*36.2*
	n/a	16	*20.8*	30	*30.9*	46	*26.4*
**Rotating place of work**	yes	12	*15.6*	23	*23.7*	35	*20.1*
	no	43	*55.8*	42	*43.3*	85	*48.9*
	n/a	22	*28.6*	32	*33.0*	54	*31.0*
**Occupational group**	nursing	33	*42.9*	43	*44.3*	76	*43.7*
	physician	0	*0.0*	2	*2.1*	2	*1.1*
	administration	3	*3.9*	10	*10.3*	13	*7.5*
	therapist	9	*11.7*	4	*4.1*	13	*7.5*
	technical staff	7	*9.1*	2	*2.1*	9	*5.2*
	social services	12	*15.6*	15	*15.5*	27	*15.5*
	others	8	*10.4*	12	*12.4*	20	*11.5*
	n/a	5	*6.5*	9	*9.3*	14	*8.0*
**Employer**	hospital	34	*44.2*	34	*35.1*	68	*39.1*
	geriatric care	33	*42.9*	40	*41.2*	73	*42.0*
	n/a	10	*13.0*	23	*23.7*	33	*19.0*

The most represented occupational group was nursing (43.7%; IG: 42.9% vs. CG 44.3%). Thus, we divided the sample into “nursing” and “other professional groups” including those who did not specify profession (n/a) for further analysis. Additionally, we divided the sample into those “working in hospital” (*n* = 68, 39.1%) and those “working in inpatient geriatric care” (*n* = 73, 42%). In the IG 44.2% and 35.2% in the CG worked full-time at which 26.8% in the CG did not specify. Moreover, 48.3% of the participants reported weekend-working and late- as well as nightshifts (37.4%) and 17.8% reported caring for relatives or children.

### Primary and secondary outcomes at baseline

The primary outcome “job satisfaction” at baseline (Table 4) was 56.58 ± 16.67. Comparing the IG and CG, there were no differences regarding COPSOQ outcomes. There was a significant difference between IG and CG in personal burnout with the IG reporting higher levels of personal burnout than the CG (58.4 ± 21.17 vs. 50.88 ± 22.85; *p* = .047).

Considering the small number of men (*n* = 18), women (*n* = 118) reported higher demands at work than men (66.6 ± 21.08 vs. 57.41 ± 24.9). At the same time, values for thoughts about quitting job were lower in women (73.26 ± 26.84 vs. 83.33 ± 12.86). Regarding balance between work and private life, nursing staff showed higher values than the other occupational groups (55.20 ± 22.56 vs. 44.20 ± 21.52).

Although work-related burnout was higher in nurses than in other occupational groups, (52.66 ± 19.19 vs. 46.94 ± 21.03) perceived work ability was found to be slightly higher in nurses.

Those with longer work experience reported a lower overall job satisfaction (*p* =.001, R^2^ = 0.059), a lower level of development opportunities (*p* =.015, R^2^ = 0.028) and perceived work ability (*p* =.009, R^2^ = 0.046). Additionally, they showed higher work-related levels of burnout (*p* =.014, R^2^ = 0.029) and emotional requirements (*p* =.007, R^2^ = 0.038).

**Table 4:** COPSOQ categories for IG and CG at baseline

Outcomes	group	min.	max.	x̄	SD
**COPSOQ category B1** **emotional requirements ** (0 low − 100 high)	*n* = 139	8.33	100.00	65.35	22.12
	IG (*n* = 67)	16.67	100.00	67.79	21.36
	CG (*n* = 72)	8.33	100.00	63.08	22.72
**COPSOQ category B2** **work and private life balance** (0 high − 100 low)	*n* = 138	0.00	100.00	48.82	22.56
	IG (*n* = 67)	7.14	100.00	50.84	21.58
	CG (*n* = 71)	0.00	100.00	46.91	23.42
**COPSOQ category B5** **development opportunities** (0 low − 100 high)	*n* = 139	20.83	100.00	69.73	15.84
	IG (*n* = 67)	29.17	95.83	70.86	15.09
	CG (*n* = 72)	20.83	100.00	68.68	16.54
**COPSOQ category B10** **thoughts about changing job** (0 low − 100 high)	*n* = 136	0.00	100.00	74.72	25.34
	IG (*n* = 66)	0.00	100.00	74.62	23.71
	CG (*n* = 70)	0.00	100.00	74.82	26.96
**COPSOQ category B11** **job satisfaction** (0 low − 100 high)	*n* = 138	8.25	100.00	56.58	16.67
	IG (*n* = 67)	9.43	85.86	56.71	13.27
	CG (*n* = 71)	8.25	100.00	56.45	19.44
**COPSOQ category B12** **state of health** (0 low − 100 high)	*n* = 139	20.00	100.00	60.22	19.13
	IG (*n* = 67)	20.00	90.00	59.85	18.95
	CG (*n* = 72)	20.00	100.00	60.56	19.42
**CBI 1** **work-related burnout** (0 low − 100 high)	*N* = 137	4.17	95.83	49.36	20.4
	IG (*n* = 67)	16.67	95.83	51.93	18.38
	CG (*n* = 70)	4.17	91.67	46.9	22.01
**CBI 2** **patient-related burnout** (0 low − 100 high)	*n* = 124	0.00	87.5	31.85	21.86
	IG (*n* = 61)	0.00	75.00	35.25	20.22
	CG (*n* = 63)	0.00	87.5	28.56	23.01
**CBI 3** **personal burnout** (0 low − 100 high)	*n* = 138	4.17	100.00	54.53	22.29
	IG (*n* = 67)	8.33	100.00	58.4	21.17
	CG (*n* = 71)	4.17	95.83	50.88	22.85
**WAI sum** (max. 22)	*n* = 133	5.00	22.00	16.01	4.00
	IG (*n* = 65)	5.00	21.00	15.66	3.85
	CG (*n* = 68)	5.00	22.00	16.34	4.13
**state of health (HS)** (1 low − 5 high)	*n* = 138	1.54	5.00	3.18	0.8
	IG (*n* = 67)	1.62	5.00	3.12	0.77
	CG (*n* = 71)	1.54	4.92	3.24	0.83

### Study population at follow-up (t_1_)

Table 2 shows the response rate for the return of questionnaires for both IG and CG. At t_1_, 29 participants in the IG returned questionnaires. 47 of 55 participants in the CG who returned questionnaires at t_1_ also returned questionnaires at t_0_. In total, 76 participants provided both, t_0_ and t_1_ information.

### Primary and secondary outcomes

Regarding the primary outcome job satisfaction, our hypothesis was not confirmed (Table 5). Nevertheless, job satisfaction in the intervention group increased from 55.47 ± 10.23 to 57.07 ± 11.65, while job satisfaction in the CG decreased from 56.29 ± 19.69 to 53.47 ± 20.09 (*p* >.05).

**Table 5:** Primary outcome - job satisfaction at t_1_

	group	*x̄* t_0_	SD t_0_	*x̄* t_1_	SD t_1_	*p*-value	Δt_1_-t_0_	SD Δt_1_-t_0_	*p*-value
**COPSOQ category B11** **job satisfaction** **(**0 low - 100 high)	IG n=28	55.47	10.23	57.07	11.65	**.129**	2.23	10.18	**.075**
	CG n=45	56.29	19.69	53.47	20.09	**.067**	-2.83	12.41	

In the IG, work-life-balance significantly improved from 55.89 ± 18.95 to 49.6 ± 24.11 (*p* =.033). Although there were no further significant changes, the IG improved in almost all COPSOQ categories in contrast to the CG and showed greater improvement in emotional requirements (−4.76 ± 17.33 vs. 2.3 ± 13.87; d = 0.28).

We found a significant difference between the groups regarding patient-related burnout (*p* =.027; f = 0.29) and personal burnout (*p* =.011; f = 0.31). There was a significant improvement in the IG in “personal burnout” (from 58.18 ± 19.83 to 52.93 ± 20.99; *p* =.024; d = 0.28). In contrast we observed a significant decline for “personal burnout” from 54.8 ± 22.3 to 59.15 ± 22.44; *p* =.038; d = 0.24) as well as or patient-related burnout from 33.33 ± 22.9 to 38.50 ± 21.58 (*p* =.036; d = 0.32). Table 6 shows the results for secondary outcomes at t_1_.

**Table 6:** Secondary outcomes at t_1_

Outcome	group	x̄ t_0_	SD t_0_		x̄ _t1_		SD t_1_		***p***>**-value**	x̄ t_1_-t_0_	SD t_1_-t_0_	***p*** **-value**
COPSOQ category B1 emotional requirements (0 low − 100 high)	IG *n* = 28	73.21	15.93		68.45		17.32		**0.079**	−4.76	17.33	**0.056**
	CG *n* = 47	64.72	20.50		67.02		20.11		**0.130**	2.30	13.87	
COPSOQ category B2 work and private life balance (0 high − 100 low)	IG *n* = 28	55.89	18.95		49.6		24.11		**0.033**	−6.29	17.35	**0.083**
	CG *n* = 46	50.22	24.08		51.55		23.3		**0.314**	1.33	18.51	
COPSOQ category B5 development opportunities (0 low − 100 high)	IG *n* = 28	67.71	12.03		68.87		12.71		**0.282**	1.16	10.49	**0.752**
	CG *n* = 47	68.05	16.82		68.26		18.11		**0.457**	0.21	13.55	
COPSOQ category B10 thoughts about changing job (0 low − 100 high)	IG *n* = 28	70.54	20.75		72.32		20.79		**0.350**	2.31	24.03	**0.946**
	CG *n* = 46	72.01	28.77		73.91		25.8		**0.310**	1.9	25.82	
COPSOQ category B12 state of health (0 low − 100 high)	IG *n* = 28	56.3	18.43		58.15		17.11		**0.247**	1.85	13.88	**0.558**
	CG *n* = 45	58.22	19.34		57.78		15.94		**0.432**	−0.44	17.18	
CBI 1 work-related burnout (0 low − 100 high)	IG *n* = 28	53.42	17.31		50.65		18.06		**0.176**	−2.77	15.49	**0.208**
	CG *n* = 45	50.46	21.47		52.83		21.99		**0.185**	2.37	17.56	
CBI 2 patient-related burnout (0 low − 100 high)	IG *n* = 28	38.67	16.38		34.77		19.13		**0.061**	−3.9	12.16	**0.027**
	CG *n* = 39	33.33	22.90		38.50		21.58		**0.036**	5.17	17.43	
CBI 3 personal burnout (0 low − 100 high)	IG *n* = 27	58.18	19.83		52.93		20.99		**0.024**	−5.25	13.1	**0.011**
	CG *n* = 46	54.80	22.31		59.15		22.44		**0.038**	4.35	16.24	
WAI sum (max. 22)	IG *n* = 26	14.77	4.07		15.58		3.74		**0.114**	0.81	3.33	**0.285**
	CG *n* = 43	16.00	3.98		15.86		4.19		**0.402**	−0.14	3.66	
State of health (HS) (1 low − 5 high)	IG *n* = 26	3.10	0.76		3.13		0.76		**0.389**	0.03	0.48	**0.218**
	CG *n* = 46	3.11	0.72		2.98		0.74		**0.053**	−0.13	0.52	

### Subgroup analyses at t_1_

Due to the insufficient number of cases, we only conducted a few subgroup analyses to understand whether effects may differ between subgroups.

Within the IG, analysis for change in job satisfaction stratified by age group, family status, occupational group and work experience showed no significant differences. However, we observed a greater improvement for work-related burnout in nursing staff in geriatric care (*n* = 17) compared to nursing staff in hospitals (*n* = 9), (−15.27 ± 13.5 vs. +3.28 ± 13.7; *p* =.003, d = 2.56).

Full-time professionals (*n* = 14) in the IG were more likely to change or quit their jobs three months after the intervention than part-time professionals (*n* = 13), (11.61 ± 22.18 vs. −7.69 ± 22.56; *p* =.034, d = 0.86).

In the CG, a higher improvement in general health status was observed for nurses (*n* = 18, from 6.67 ± 18.15) versus other occupational groups (*n* = 27, 3.7 ± 15.48; *p* =.046, d = 0.63).

### Study population at follow-up (t_2_)

Due to the limited data available at t2, we only provide selected results. Table 7 shows the percentage for those who completed baseline and follow-up evaluation and those who did not.

**Table 7:**
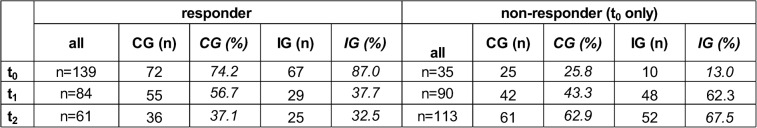
Responders and non-responders at t_0_, t_1_ and t_2_

### Primary and secondary outcomes at t_2_

Overall job satisfaction in the IG changed from 54.05 ± 10.60 at t_0_ to 57.07 ± 11.65 at t_1_ and resulted in 54.12 ± 11.86 at t_2_. Thus, job satisfaction in the IG after six months was about baseline-level whereas job satisfaction in the CG continually declined and significantly decreased below baseline (−5.67 ± 12.69; *p* =.009; *p* =.009, Table 8).

**Table 8:** Primary outcome - job satisfaction at t_2_

	**group**	***x̄*** ** t**_**0**_	**SD** **t**_**0**_	***x̄*** **t**_**2**_	**SD** ** t**_**2**_	***p*** **-value**	**x̄ t**_**2**_-t_**0**_	**SD** ** t**_**2**_ **-t**_**0**_	***p*** **-value**
**COPSOQ** **category** **B11job satisfaction **(0 low – 100 high)	IG *n*=23	54.05	10.60	54.12	11.86	**.487**	0.06	9.53	**.075**
	CG *n*=31	58.58	21.51	52.91	16.52	**.009**	-5.67	12.69	

After six months, no considerable changes were found both in the IG and the CG and between groups. Emotional requirements as well as thoughts about changing the job assessed by the COPSOQ-questionnaire still showed better scores in the IG whereat other category scores in the IG converged to CG scores at 6 months. Table 9 shows secondary outcomes at t_2_:

**Table 9:** Secondary outcomes at t_2_

**Outcome**	**group**	**x̄t**_**0**_	**SD t**_**0**_	**x̄t**_**2**_	**SD t**_**2**_	***p*** **-value**	**x̄ t**_**2**_ **-t**_**0**_	**SD t**_**2**_ **-t**_**0**_	***p*** **-value**
**COPSOQ category B1 emotional requirements **(0 low - 100 high)	IG *n*=22	70,45	16,21	67,05	20,81	**.217**	−3.41	20.03	**.281**
	CG *n*=34	67,89	20,11	70,1	21,23	**.240**	2.21	18.04	
**COPSOQ category B2 work and private life balance ** (0 high - 100 low)	IG *n*=23	57,19	18,0	52,64	25,5	**.130**	−4.55	18.93	**.252**
	CG *n*=32	50,73	21,93	51,28	22,51	**.411**	0.55	13.82	
**COPSOQ category B5development opportunities** (0 low - 100 high**)**	IG *n*=23	65,94	13,16	63,22	12,97	**.148**	−2.72	12.15	**.931**
	CG *n*=34	68,09	17,17	65,07	18,0	**.092**	−3.01	12.95	
**COPSOQ category B10 thoughts about changing job** (0 low - 100 high)	IG *n*=20	73,75	16,67	68,13	23,81	**.113**	−5.63	20.06	**.405**
	CG *n*=32	75,39	27,22	75,39	23,22	**.500**	0	25.40	
**COPSOQ category B12 state of health** (0 low - 100 high)	IG *n*=23	54,78	18,55	57,83	17,31	**.187**	3.04	16.08	**.721**
	CG *n*=34	62,65	19,43	64,12	15,4	**.302**	1.47	16.35	
**CBI 1 work-related burnout** (0 low - 100 high)	IG *n*=23	55,43	19,44	53,80	21,94	**.321**	−1.63	16.56	**.480**
	CG *n*=31	49.06	22,28	50,40	19,32	**.300**	1.34	14.13	
**CBI 2 patient-related burnout** (0 low - 100 high)	IG *n*=20	39,79	15,79	36,29	23,41	**.243**	−3.50	22.07	**.470**
	CG *n*=30	29,64	20,17	30,0	19,69	**.450**	0.36	15.48	
**CBI 3** **Personal burnout** (0 low - 100 high)	IG *n*=23	59,42	20,69	60,51	27,0	**.372**	1.09	15.80	**.960**
	CG *n*=33	52,4	24,3	53,28	24,82	**.361**	0.88	14.16	
**WAI sum ** (max. 22)	IG *n*=22	14.5	4,34	14,77	3,73	**.364**	0.27	3.63	**.382**
	CG *n*=31	16,55	4,17	16,03	3,65	**.163**	−0.52	2.87	
**status of health ** (1 low - 5 high)	IG *n*=23	5,48	1,86	5,78	1,73	**.187**	0.3	1.61	**.721**
	CG *n*=34	6,26	1,94	6,41	1,54	**.302**	1.47	1.64	

## Discussion

Healthcare professionals, especially nurses, experience a multitude of work related stressors, which affect physical and mental well-being [1, 2]. In addition, traumatic experiences can induce burnout or PTSD symptoms [9, 10, 16]. Numerous studies underline the importance of implementing health promotion interventions and the need to investigate its effectiveness on work-related health in at-risk occupations [33–35]. The main purpose of our study was to evaluate the effectiveness of CSG on job satisfaction and work-related stress. We designed the intervention based on creative art therapy, which uses art-based activities to treat emotional and mental health conditions [47].

Regarding mindfulness based interventions; evidence shows that changes in mindfulness are linked to better physical and psychological outcomes [48]. The authors assume that creative art therapy elements on the lines of mindfulness-based interventions foster a non-judgmental awareness of the present moment, which helps individuals to recognize and label their emotions without becoming overwhelmed or reactive. By observing and dealing with emotions as they arise, people can manage stress more effectively [49].

The CSG supported this shift towards acceptance, which led to a decrease in stress and burnout levels and greater emotional balance. A major part of the CSG was the emphasis on self-compassion, where individuals learned to treat themselves with kindness and patience, especially in moments of strain. This shift from self-criticism to self-kindness might have reduced feelings of stress and helped to buffer the impact of stressful events [49]. Mindfulness improves cognitive flexibility, allowing individuals to shift their perspective on stressors. Rather than seeing challenges as insurmountable, they learn to view them as temporary and manageable. This shift in cognitive patterns helps to reduce the perceived intensity of stress and anxiety.

Therefore, the CSG might have mediated job satisfaction, stress and burnout levels through a combination of cognitive, emotional, and physiological mechanisms and enhanced the ability to cope with challenges in a more adaptive, resilient manner.

## Results

Overall job satisfaction at baseline was found to be 56.89 ± 16.53 and thereby lower than compared to the German reference population [48]. This finding aligns with results from Peter et al. [49], showing that health professionals in hospitals as well as those working in nursing homes showed higher burnout symptoms and lower job satisfaction.

Regarding our study sample, there are several reasons to come into consideration: First, reference values were investigated almost twenty years ago when working conditions in the healthcare sector were not as bad as today. Secondly, the study focused on healthcare professionals perceiving high levels of work-related stress. Thirdly, the increasing workload and the pandemic restrictions [50] contributed to even higher levels of work-related strain and stress [51].

Our results indicate that CSG are effective in improving job satisfaction and perceived work-related burdens. Even though the effect was not statistically significant, participants of the IG reported an increase in job satisfaction. While job satisfaction increased from 55.47 ± 10.23 to 57.07 ± 11.65 in the IG, we found a decrease in the CG from 56.29 ± 19.69 to 53.47 ± 20.09 at t_1_. Correspondingly, participants of the IG experienced improvements in perceived work-life-balance and a significant improvement in patient-related burnout compared to the CG, indicating that they adopted effective coping strategies to reduce stress. We assume that the special conditions of the pandemic have influenced the results, but not in the sense of changing the direction of the effects.

Notably, improvements in the primary as well as secondary outcomes declined between month three and month six, which is in line with other findings [52]. After six months, the IG had reached baseline job satisfaction levels again; whereas levels of job satisfaction in the CG continued to decrease below baseline.

### Limitations and strengths

It is essential to acknowledge the limitations of this study, particularly the sample size and loss to follow-up, limiting the analytic generalization to provide evidence of a causal link for subgroups or individuals. Furthermore, conducting the study during the Covid-19 pandemic was challenging. The pandemic worsened working conditions for healthcare professionals and affected recruitment, participation and delivery of intervention. Our long questionnaire based on the referred assessments might have implied an important respondent’s drop-off during the questionnaire completion. Furthermore, staying away from work for two days leads to staff-shortage, which may induce bad conscience or add additional stress so that participants desisted from attending the course. According to a study by Otto et al. [53], 41% of outpatient and 45% of inpatient carers reported not having enough time or do not wish to participate in interventions beyond the working day. Our CSG-schedule was inter-coordinated with the participating institutions who concordantly preferred two longer sessions rather than more for example shorter weekly courses. Since the participants did not have any creative arts therapy- experience and because of the nature of the CSG, emphasizing the participant-participant interaction as well participant-therapist interaction we desisted from conducting online sessions. We assume that, mainly due to the overall workload and the pandemic restrictions [51, 54], participants of the IG did not continually participate in the intervention and did not complete all follow-up assessments.

Although 196 participants were initially included, the number available for analysis was very low due to dropout and loss to follow-up. Because of the remarkable number of missing baseline data, we decided to analyse available data only for those who completed baseline as well as the study endpoints. Thereby, we were able to assess the effectiveness of the intervention for those who participated in the IG, knowing that effectiveness of the intervention may be overestimated, sample size attenuated, the statistical power of the study decreased and benefits of randomization may be affected [55].

Another point is that physicians are underrepresented relative to nursing staff in our study as well as in current research [17]. Although we tried to address different occupational groups by including hospital staff, it was almost impossible to include physicians.

### Implications on health systems

Improving nurses’ job satisfaction and well-being are not only personal challenges concerning those experiencing particularly high levels of stress but also an important concern with broad implications for the health system. Satisfied nurses are more engaged and motivated, which leads to higher quality of care and patient satisfaction. Pentescu et al. [56] showed that more satisfied patients are more compliant. Again, considering that compliance is associated with faster convalescence, improvement of nurses job satisfaction and prevention of emotional stress and burn out are key factors for quality of care and patient satisfaction [57, 58]. Again, quality of care and patient satisfaction are positively associated with nurses’ levels of job satisfaction [59]. Low levels of job satisfaction as well as high levels of burn out contribute to increased turnover rates among nurses, which leads to higher recruitment and training costs for healthcare organizations and can result in a shortage of experienced staff, putting additional strain on remaining team members. The financial implications of burnout therefore are substantial [60].

## Conclusion

The aim of the intervention was to provide coping strategies that improve job satisfaction and prevent work-related health problems.The findings of our study strengthen the idea that health promotion interventions based on creative art therapy can reduce emotional requirements, patient-related burnout and personal burnout. Addressing structural items such as development opportunities and work-related burnout, we did not observe any improvement. We must take into account that the impact of individual interventions in general is less effective compared to structural or combined interventions [61, 62]. Those changes require systemic changes, including improving work conditions, providing adequate support and resources, promoting work-life balance, and implementing effective interventions to manage stress [63]. As long as nursing professions in Germany are facing bad working conditions based on inefficiencies in the German health system [51], it remains difficult to support their resources by health promotion interventions and to investigate effectiveness in research.

Future research should explore the accessibility of people employed in the healthcare sector and their willingness to participate in intervention studies. Besides participation barriers in general, studies should focus on the factors that address physicians’ non-participation as well as structural prevention strategies and initiatives to strengthen physician engagement. However, further studies should evaluate the potential of creative arts therapy in occupational health promotion as well as its underlying mediators and mechanisms.

## Data Availability

The data generated and analysed during the current study supporting the findings are available on request from the corresponding author. The data are not publicly available due to privacy and ethical restrictions.

## Abbreviations

CBI: Copenhagen Burnout inventory
COPSOQ: Copenhagen psychosocial questionnaire
CSG: Creative Strengthening Groups
IIT: Intention-to-treat
PTSD: Post traumatic stress disorder
WAI: Work Ability Index
